# Not Only *Leptotrombidium* spp. an Annotated Checklist of Chigger Mites (Actinotrichida: Trombiculidae) Associated with Bacterial Pathogens

**DOI:** 10.3390/pathogens11101084

**Published:** 2022-09-23

**Authors:** Hanna Moniuszko, Konrad Wojnarowski, Paulina Cholewińska

**Affiliations:** 1 Section of Basic Research in Horticulture, Department of Plant Protection, Institute of Horticultural Sciences, Warsaw University of Life Sciences—SGGW, 02-776 Warsaw, Poland; 2 Chair for Fish Diseases and Fisheries Biology, Ludwig-Maximilians-University of Munich, 80539 Munich, Germany; 3 Institute of Animal Breeding, Wroclaw University of Environmental and Life Sciences, 51-630 Wroclaw, Poland

**Keywords:** *Anaplasma*, *Bartonella*, *Borrelia*, *Coxiella*, *Francisella*, *Leptospira*, *Mycobacterium*, *Orientia*, *Rickettsia*

## Abstract

Mites of the family Trombiculidae are known for playing a role in maintaining and spreading the scrub typhus etiologic agent, an intracellular Gram-negative bacterium, *Orientia tsutsugamushi*. Species of the genus *Leptotrombidium* are investigated most thoroughly, particularly in SE Asia, and a few are proven vectors for the pathogen. The mentioned association, however, is not the only one among trombiculids. Here, we present a list of chiggers indicated in the literature as positive for bacterial pathogens, tested throughout almost 100 years of research. Taxonomic identities of trombiculids follow recent revisions and checklists. Results point at 100 species, from 28 genera, evidenced for association with 31 bacterial taxa. Pathogen-positive mites constitute around 3.3% of the total number of species comprising the family. Discussed arachnids inhabit six biogeographic realms and represent free-living instars as well as external and internal parasites of rodents, soricomorphs, scadents, lagomorphs, peramelemorphs, bats, passerine birds, reptiles and humans. A variety of so far detected bacteria, including novel species, along with the mites’ vast geographical distribution and parasitism on differentiated hosts, indicate that revealing of more cases of Trombiculidae-pathogens association is highly probable, especially utilizing the newest techniques enabling a large-scale bacterial communities survey.

## 1. Introduction

Trombiculidae, as understood by Kudryashova [1] (Actinotrichida: Parasitengona), comprise 3013 species inhabiting seven zoogeographic regions [2]. These mites are known for the complex life cycle consisting of egg, prelarva, obligatorily parasitic larva, calyptostatic protonymph, predatory deutonymph, calyptostatic tritonymph and predatory adult form [3]. Parasitic instars are especially abundant on small mammals—rodents, insectivores and bats, yet, parasitism of birds, reptiles and amphibians is also reported. Feeding on bigger animals, such as carnivores, ungulates and lagomorphs, occurs considerably less frequently. The rarest cases regard parasitizing invertebrates [4,5,6,7,8,9]. Humans are considered accidental hosts for Trombiculidae, however, locally (especially in SE Asia, often on so-called ‘chigger islands’) parasitism on people is a frequent phenomenon. Recorded duration of larval attachment to a human’s body ranged from one to three days [10,11].

Trombiculid larvae feed basically on dissolved connective tissue similar in composition to a plasma, however, in single cases, ingestion of blood has been also reported [12,13,14]. For the purpose of food intake, after reaching a parasitope, mites pierce the host’s skin with chelicerae and, alternately, secrete enzymes, digesting epithelium and protein substances, solidifying in contact with host tissues. As a result, a stylostome is formed—a canal with a strengthened sidewall, linear or widened distally. This channel is successively extended until the cessation of the parasitic phase [12,15,16]. Such a feeding mechanism implies the group’s medical-veterinary importance, comprising of two issues—inducing, relatively harmless but bothersome (intensive itching), local skin inflammations (called *trombiculiasis*, *trombiculosis* or *erythema autumnale*) being the immunological reaction of the host’s body to larval lytic secretions, and the capability of acquiring and spreading bacterial pathogens, the source of which being the vertebrate organisms [16,17,18].

Among Trombiculidae, representatives of *Leptotrombidium* spp. are best recognized for maintaining and transmitting (to the hosts, as well as transstadially and transovarially—to the offspring) an etiologic agent of scrub typhus, intracellular bacterium, *Orientia tsutsugamushi* (Hayashi, 1920) (Pseudomonadota, Rickettsiales, Rickettsiaceae), formerly under the names of *Rickettsia tsutsugamushi* and *R. orientalis* [19,20,21]. Due to the significance of the illness, involving lethality without treatment, and its high prevalence in the endemic areas of SE Asia, the relation *Leptotrombidium* spp.—*O. tsutsugamushi* is most well explored in literature. Issues raised so far concern scrupulously investigated ecological, epidemiological, molecular and geographical aspects of the chigger-borne rickettsiosis occurrence in general as well as in particular areas of China, South Korea, Japan, Russia, Taiwan, Thailand and Asia–Australia–Pacific region, which together constitute the so called ‘tsutsugamushi triangle’ [10,14,22,23,24,25,26,27,28,29,30,31,32,33,34,35]. Recent studies, however, indicate that scrub typhus is no longer limited to the above-mentioned zone, as cases from Africa, Middle East and South America have been also reported [36,37,38,39].

Despite focusing the research on *O. tsutsugamushi*, it is not the only pathogenic bacterium associated with trombiculid mites. Records considering other bacterial taxa represent, however, mostly single cases and are rather scattered in the literature. As for the broad scale studies on bacteria and parasitic mites, Chaisiri et al. provided new data on the bacterial flora associated with chiggers in Thailand and summarized the published information on microbiome of representatives of Sarcoptiformes, Trombidiformes and Mesostigmata orders [40,41]. Herrera-Mares et al., in turn, recently reviewed the knowledge on the ecology of infectious and parasitic diseases being shaped by the interactions between parasites representing Trombidiformes and Mesostigmata, their rodent hosts and related etiological agents occurring worldwide [42].

The aim of creating the present review was to provide the most actual list of valid trombiculid taxa, naturally infected with bacterial pathogens, together with information on hosts from which mites were collected (provided it was possible or relevant) and countries of records, reported in the published sources.

## 2. Results

### 2.1. Valid Trombiculid Taxa Associated with Bacterial Pathogens

**Genus: *Acomatacarus* Ewing, 1942**

***Acomatacarus* sp.**

Mites of this genus are known for the association with *O. tsutsugamushi* revealed during research in China. Infected larvae were collected from the lesser ricefield rat *Rattus losea* (Swinhoe, 1871), the brown rat *R. norvegicus* (Berkenhout, 1769), the black rat *R. rattus* (Linnaeus, 1758)*,* the house mouse *Mus musculus* (Linnaeus, 1758), *M. bactrianus kakhyenensis* (most probably the Ryukyu mouse *M. caroli* Bonhote, 1902) (Mammalia: Rodentia) and the Asian house shrew *Suncus murinus* (Linnaeus, 1766) (Mammalia: Soricomorpha) [14,43,44,45].

**Genus: *Ascoschoengastia* Ewing, 1948**

***Ascoschoengastia* spp.**

Undetermined to the species level representatives of the genus are known to harbor *Bartonella* spp. Strong et al., 1915 (Pseudomonadota, Hyphomicrobiales, Bartonellaceae), *O. tsutsugamushi* and *Rickettsia* sp. da Rocha-Lima, 1916 (Pseudomonadota, Rickettsiales, Rickettsiaceae). In the case of the latter two, a co-infection was observed in Thailand. Tested chiggers parasitized the Asian house rat *R. tanezumi* Temminck, 1844, *R. rattus*, *R. norvegicus, R. rattus*-complex (i.e., *R. tanezumi, R. losea sakeratensis* Gyldenstolpe, 1917, *Rattus* sp.), the Savile’s bandicoot rat *Bandicota savilei* Thomas, 1916, the greater bandicoot rat *B. indica* (Bechstein, 1800) (Mammalia: Rodentia), the northern treeshrew *Tupaia belangeri* (Wagner, 1841) and the common treeshrew *T. glis* Diard and Duvaucel, 1820 (Mammalia: Scadentia). Reports come from India, Thailand and Vietnam [23,30,46,47,48,49,50].

***A. audyi* (Womersley, 1952)**

Individuals of the species were positive for the presence of *O. tsutsugamushi*. Collected in the Malayan forest [14].

***A. indica* (Hirst, 1915)**

Species mentioned also as *Euschoengastia indica*. Associated with *O. tsutsugamushi* and *R. typhi* (Wolbach and Todd, 1920). Positive larvae fed on rats, including the ricefield rat *R. argentiveter* (Robinson and Kloss, 1916) and squirrel. Collected and tested in China, Indonesia, Malaysia, Thailand and Vietnam [44,51,52,53,54].

**Genus: *Blankaartia* Oudemans, 1911**

***Blankaartia* spp.**

In chiggers from this genus, the following pathogens were detected: *Bartonella* spp., *B. tamiae* Kosoy et al., 2008 and *O. tsutsugamushi*. Host animals included rats from *R. rattus*-complex, *R. rattus*, *R. tanezumi*, *R. argentiventer*, *B. indica*, *B. savilei* and the fawn-colored mouse *M. cervicolor* Hodgson, 1845. Records originate from Thailand and Vietnam [10,48,50,55].

***B. acuscutellaris* (Walch, 1922)**

Known for association with *O. tsutsugamushi*. Infected larvae were collected from *T. glis* and rodents including *R. rattus* in Thailand [46,52].

***B. sinnamaryi* (Floch and Fauran, 1956)**

Pathogen detected in this species was described as *Rickettsia felis*-like and chiggers were collected from passerine birds: the ruby-crowned tanager *Tachyphonus coronatus* (Vieillot, 1822) and the pale-breasted thrush *Turdus leucomelas* Vieillot, 1818 (Aves: Passeriformes) in Brazil [56,57].

**Genus: *Cheladonta* Lipovsky, Crossley and Loomis, 1955**

***C. costulata* (Willmann, 1952)**

Species harboring *R. helvetica* Beati et al., 1993 and *R. monacensis* Simser et al., 2002 and associated with the following bacteria-positive host rodents: the bank vole *Myodes glareolus* (Schreber, 1780), the yellow-necked wood mouse *Apodemus flavicollis* (Melchior, 1834), the common vole *Microtus arvalis* (Pallas, 1778) and the European wood mouse *A. sylvaticus* (Linnaeus, 1758) (Mammalia: Rodentia). Mites captured in Slovakia [58].

***C. ikaoensis* (Sasa, Sawada, Kanoh, Hayashi and Kumada, 1951)**

Associated with *O. tsutsugamushi* and collected from field rodents, mostly the large Japanese field mouse *A. speciosus speciosus* (Temminck, 1844), the small Japanese field mouse *A. argenteus argenteus* (Temminck, 1844), *Eothenomys kageus* Imaizumi, 1957 (Mammalia: Rodentia) and the Japanese grass vole *M. montebelli montebelli* (Milne-Edwards, 1872) in Japan [59,60].

**Genus: *Ericotrombidium* Vercammen-Grandjean, 1965**

***E. jayewickremei* (Womersley, 1952)**

*Orientia tsutsugamushi*-positive larvae of this species (originally mentioned as *Leptotrombidium jayawickremei*) were captured in India while feeding on *R. rattus* [49].

**Genus: *Euschoengastia* Ewing, 1938**

***Euschoengastia* sp.**

Individuals of the genus collected from Malayan jungle rats tested positive for *O. tsutsugamushi* [61].

***E. koreaensis* Jameson and Toshioka, 1954**

*Orientia tsutsugamushi*-positive larvae were collected from *A. agrarius*, the Korean red-backed vole *M. regulus* (Thomas, 1907) and the Ussuri white-toothed shrew *Crocidura lasiura* Dobson, 1890 (Mammalia: Soricomorpha) in South Korea [34,62,63].

**Genus: *Eutrombicula* Ewing, 1938**

***Eutrombicula* spp.**

Individuals of this genus tested positive for sequences of *Rickettsia* sp., and species very closely related to *R. conorii*, *R. felis* and *R. typhi*. Larvae were collected from birds in Brazil and from the hispid cotton rat *Sigmodon hispidus* Say and Ord (Mammalia: Rodentia), 1825 in the USA (North Carolina) [57,64].

***E. alfreddugesi* (Oudemans, 1910)**

*Rickettsia bellii*-like sequence was detected in isolates obtained from larvae of the common Northamerican chigger parasitizning snake *Philodryas nattererii* Steindachner, 1870 (Reptilia: Squamata) in Brazil [65]. 

Remarks: According to Sajle [66], *R. bellii* represent a non-pathogenic ancestral group within Rickettsiaceae, not the typhus or the spotted fever group. On the other hand, the species is evidenced to elicit an immune response in capybaras and horses, therefore, we included this association. Mechanisms of *R. bellii* possible pathogenicity, however, require more research [67].

***E. tinami* (Oudemans, 1910)**

Species evidenced to harbor a novel bacterium *Candidatus* Rickettsia colombianensi. Captured parasitizing the Andean sparrow *Zonotrichia capensis* (Müller, 1776) and *T. coronatus* in Brazil (Aves: Passeriformes) [57].

***E. wichmanni* (Oudemans, 1905)**

Host-questing larvae of the species, collected with black plates, were reported to test positive for *O. tsutsugamushi* in Thailand [68].

**Genus: *Gahrliepia* Oudemans, 1912**

***Gahrliepia* sp.**

Known for the association with *O. tsutsugamushi* and *Rickettsia* sp. Collected from *R. rattus*-complex, *R. tanezumi*, *B. savilei*, *B. indica* and *T. belangeri*. Records come fromChina and Thailand [30,45,47,50].

***G. saduski* Womersley, 1952**

Unengorged and parasitic (on *A. speciosus*) larvae captured in Japan tested positive for *O. tsutsugamushi* [69,70].

***G. xiaowoi* Wen and Xiang, 1984**

Individuals of the species contained *O. tsutsugamushi*. Material collected from the Bower’s white-toothed rat *Berylmys bowersi* (Anderson, 1879) (Mammalia: Rodentia) in Thailand [23].

**Genus: *Helenicula* Audy, 1954**

***Helenicula* sp.**

Genus listed among mites positive for *O. tsutsugamushi* and *Rickettsia* sp., parasitizing *R. tanezumi*, *B. savilei* and *B. indica* in Thailand [47].

***H. miyagawai* (Sasa, Kumada and Miura, 1951)**

Species individuals (also reported as *Euschoengastia miyagawai*) captured with chigger traps and collected from rodents (mainly *A. agrarius*) revealed the presence of *O. tsutsugamushi* in South Korea and some other *Rickettsia* species (most probably not *O. tsutsugamushi*) in Japan [14,34,71,72].

***H. naresuani* Stekolnikov, 2016**

Associated with *O. tsutsugamushi*. Captured on *T. glis* in Thailand [24].

**Genus: *Herpetacarus* Vercammen-Grandjean, 1960**

***H. antarctica* (Stekolnikov and Gonzalez-Acuña, 2015)**

The species is a proven vector for *Candidatus* Orientia chiloensis Abarca et al. 2020, a novel bacterium causing scrub typhus in the area of subantarctic Chile. Parasitic mites were collected from human hosts while unengorged individuals from low vegetation [73].

***H. eloisae* Stekolnikov and Silva-de la Fuente, 2021**

*Orientia* spp-positive chiggers fed on the olive-colored akodont *Abrothrix olivacea* (Waterhouse, 1837), the Sanborn’s akodont *A. sanborni* (Osgood, 1943) and the Valdivian long-clawed akodont *Geoxus valdivianus* (Philippi, 1858) (Mammalia: Rodentia) in Chile [73,74,75].

***H. hertigi* (Brennan, 1970)**

A species reported to carry a novel bacterium *Candidatus* Rickettsia colombianensi. Larvae were collected from the colilargo *Oligoryzomys* sp. Bangs, 1900 (Mammalia: Rodentia) in Brazil [76].

**Genus: *Hirsutiella* Schluger and Vysotzkaja, 1970**

***H. zachvatkini* (Schluger, 1948)**

Species tested positive for *R. helvetica* and *R. monacensis.* Fed on *Rickettsia*-positive rodents: *M. glareolus*, *A. flavicollis*, *M. arvalis* and *A. sylvaticus,* captured in Slovakia [58].

**Genus: *Intercutestrix* Brennan and Yunker, 1966**

***I. mondolfi* Brennan and Yunker, 1969**

Species associated with *Coxiella burnetii* (Derrick, 1939) (Pseudomonadota, Legionellales, Coxiellaceae). Larvae were attached to the nasal cavities of the Central American spiny rat *Proechimys semispinosus* (Tomes, 1860) (Mammalia: Rodentia) in Panama [77].

**Genus: *Leptotrombidium* Nagayo, Miyagawa, Mitamura and Imamura, 1916**

***Leptotrombidium* spp.**

Representatives of *Leptotrombidium* spp. were reported to carry *B. tamiae*, *O. tsutsugamushi* and *Rickettsia* sp. Infected chiggers parasitized small mammals: *R. rattus*, *R. rattus*-complex, *R. argentiventer*, *R. tanezumi*, the Polynesian rat *R. exulans* (Peale, 1848), *M. cervicolor*, *B. indica*, *B. saliviei*, the Royle’s mountain vole *Alticola roylei* (Gray, 1842) (Mammalia: Rodentia) and *T. belangeri* as well as swarmed on the ground and plants (unfed parasites). Above observations come from India, Indonesia, Pakistan, Taiwan and Thailand [29,30,47,49,50,55,68,78,79].

***L. akamushi* (Brumpt, 1910)**

Species listed among the most important *O. tsutsugamushi* vectors. Positive larvae were obtained from *Rattus* spp., *R. exulans*, as well as from the vegetation. Records come from Japan, Malaysia, New Guinea, Philippines and Solomon Islands [21,33,54,60,80].

***L. arenicola* Traub, 1960**

*Orientia tsutsugamushi*-infected larvae were collected from *Rattus* spp. and from the ground in Indonesia and Malaysia [81,82].

***L. arvinum* (Schluger, Grokhovskaya, Dang-Van-Ngu, Nguen-Xuan-Hoe and Do-Kinh-Tung, 1960)**

Host-seeking larvae collected from the black plates tested positive for *O. tsutsugamushi* in Thailand [68].

***L. bodense* (Gunther, 1940)**

Unengorged larvae obtained with the above-mentioned technique were positive for *O. tsutsugamushi*. Malaysia [83].

***L. chaigraiensis* Tanskul and Linthicum 1997**

Species listed among proven vectors of *O. tsutsugamushi*. Infected larvae were taken from the bodies of *R. losea* and *R. rattus* captured in Thailand [84,85].

Remarks: According to revision of *Leptotrombidium* genus by Stekolnikov [86] and check list of Asian trombiculids by Chaisiri et al. [87], *L. chiangraiensis* should be considered a synonym of *L. imphalum*, as metric traits of both taxa overlap. Still, molecular sequences subsequently provided by Kumlert et al. [88] indicated distinctiveness of the two species, therefore, we treat the data for *L. chaigraiensis* separately.

***L. deliense* (Walch, 1922)**

The species is most frequently listed (also as *L. deliensis*) as a proven and widespread vector of *O. tsutsugamushi*, however, it is also reported for the association with *Borrelia* spp. Swellengrebel, 1907 (Spirochaetota, Spirochaetales, Borreliaceae), *Rickettsia* sp., species close to *R. australis*, *R. felis* Bouyer et al., 2001, *R. conorii* Brumpt, 1932, *R. raoultii* Mediannikov et al., 2008, *R. rhipicephali* Burgdorfer et al., 1978 and *R. typhi*, as well as a novel species *Candidatus* Rickettsia jingxinensis. Bacteria-positive larvae were obtained from *A. agrarius*, the Indian mole-rat *B. bengalensis* Gray, 1835, *B. indica*, *A. roylei*, *R. rattus*, the buff-breasted rat *R. tanezumi flavipectus* (Milne-Edwards, 1872), *R. tanezumi*, the Sikkim rat *R. andamanensis* (Blyth, 1860), *R. exulans*, *R. norvegicus*, *R. losea*, *Rattus* spp., the small white-toothed rat *B. berdmorei* (Blyth, 1851), *T. belangeri* (mentioned as *T. belangeri persurae*), *T. glis* and *S. murinus* (mentioned as *S. murinus fulvo-cinerea)* as well as from the moist marshlands (host-questing larvae). The above observations were made in Australia, China, India, Indonesia, Malaysia, Myanmar, Pakistan, Papua New Guinea, Philippines, Singapore, Taiwan (including Pescadores), Thailand and Vietnam [10,14,23,40,46,49,51,52,68,78,83,89,90,91,92,93,94,95,96,97,98,99,100,101,102].

***L. dihumerale* Traub and Nadchatram, 1967**

Mentioned among *O. tsutsugamushi* infected mites in India and Pakistan [14,103].

***L. fletcheri* (Womersley and Heaslip, 1943)**

Parasitic larvae feeding on the common echymipera *Echymipera kalubu cockerelli* (Ramsay, 1877) (Mammalia: Peramelemorphia) as well as questing ones taken from the ground in Papua New Guinea, Philippines and Malaysia tested positive for *O. tsutsugamushi* [14,83,104].

***L. fujii* (Kuwata, Berge and Philip, 1950)**

Species mentioned as positive for *O. tsutsugamushi.* Infected larvae parasitized *A. speciosus* and unengorged chiggers were collected from ground and vegetation in Japan [69,70]. 

***L. gaohuense* Wei, Tong and Shi, 1987**

Listed (as *L. gaohuensis*) in *Epidemiology and ecology of rickettsial diseases in the People’s Republic of China* as a vector of *O. tsutsugamushi* [44].

***L. himizui* (Sasa, Kumada, Hayashi, Enomoto, Fukuzumi and Obata, 1951)**

*Orientia tsutsugamushi*-associated species (originally listed as *L. himizu*). Host-seeking larvae were gathered from the ground and vegetation in Japan [69].

***L. imphalum* Vercammen-Grandjean and Langston, 1976**

Species known to carry *O. tsutsugamushi*, *R. conorii*, *R. typhi*, *Rickettsia* sp. and a novel bacterium *Candidatus* Rickettsia jingxinensis. Laboratory reared individuals originating from larvae captured in nature were also positive for *Mycobacterium* sp. Lehmann and Neumann, 1896 (Actinomycetota, Mycobacteriales, Mycobacteriaceae) Infected larvae were obtained from the bodies of *A. agrarius*, *B. indica*, *R. exulans*, *R. losea*, *R. rattus* and *R. tanezumi* captured in Taiwan and Thailand [23,29,84,85,97,105].

***L. insulare* Wei, Wang and Tong, 1989**

Listed among *O. tsutsugamushi* vectors in China [106].

***L. intermedium* (Nagayo, Mitamura and Tamiya, 1920)**

Species associated with *O. tsutsugamushi*. Positive larvae were obtained from *A. speciosus*, *M. musculus* and *R. norvegicus* as well as from the vegetation (prior to parasitic phase). Records come from China and Japan [70,107,108,109].

***L. kawamurai* (Fukuzumi and Obata, 1953)**

Larvae parasitizing the gray red-backed vole *M. rufocanus* (Sundevall, 1846) (originally mentioned as *Clethrionomys rufocanus bedfordiae*) and *A. speciosus ainu* were reported as *O. tsutsugamushi*-positive in Japan [81,107,110].

***L. keukenshrijveri* (Walch, 1924)**

Unengorged larvae gathered from the ground by the black plates method in Malaysia tested positive for *O. tsutsugamushi* [83].

***L. kitasatoi*** **(Fukuzumi and Obata, 1950)**

Species evidenced as positive for *O. tsutsugamushi*. Parasitic larvae were taken from *A. speciosus* and unengorged chiggers derived from the ground and vegetation in Japan [69,70,107].

***L. linhuaikongense* (Wen and Hsu, 1961)**

Known for association with *O. tsutsugamushi*. Positive parasites originated mostly from rodents: *A. agrarius*, *M. musculus*, *R. norvegicus* and the greater long-tailed hamster *Tscherskia triton* (de Winton, 1899) (Mammalia: Rodentia) captured in China. Scrub typhus agent was also detected in a nymph reared in the laboratory from engorged larva [109,110,111,112,113,114].

***L. murotoense* (Sasa and Kawashima, 1951)**

Listed among *O. tsutsugamushi* vectors in Japan [60,107].

***L. orientale* (Schluger, 1948)**

*Orientia tsutsugamushi*-positive parasitic larvae were found in South Korea and Russia (Primorsky Krai). Chiggers were captured on wild animals including *A. agrarius*, *M. regulus* and *C. lasiura* [34,62,63,115,116,117].

***L. pallidum*** **Nagayo, Miyagawa, Mitamura and Tamiya, 1919**

Species reported as positive for *O. tsutsugamushi*, *R. conorii* and *Rickettsia* sp. Infected individuals were collected from the vegetation (unengorged larvae) and the following mammal hosts: *A. agrarius*, *A. speciosus*, *B. indica*, *M. fortis*, *M. montebelli*, *M. regulus*, ‘*C. triton*’ (most probably *T. triton*), *R. exulans*, *R. losea*, *R. tanezumi* and *C. lasiura*. Reports come from Japan, Russia (Primorsky Krai), South Korea and Taiwan. [34,60,62,63,69,70,97,108,115,118,119,120].

***L. palpale* (Nagayo, Miyagawa, Mitamura and Tamiya, 1919)**

*Orientia tsutsugamushi*-positive species, larvae of which were collected from *A. agrarius*, *A. speciosus*, *M. regulus*, *M. musculus*, *R. norvegicus*, *T. triton* and *C. lasiura*. Laboratory reared nymph also tested positive for the presence of the pathogen. Records originate from China, Japan, Russia (Primorsky Krai) and South Korea [34,60,62,63,70,109,111,113,114,115,117].

***L. pavlovskyi* (Schluger, 1948)**

Species associated with *O. tsutsugamushi,* collected and examined in Russia (Primorsky Krai). Pathogen-positive individuals included parasitic larvae feeding on infected rodents and shrews: *A. agrarius*, ‘*C. triton*’ (most probably *T. triton*), the reed vole *M. fortis* (Büchner, 1889) and *C. lasiura*, along with nymphs reared in the laboratory [28,115,117,120].

***L. peniculatum* Traub and Lakshana, 1966**

Unengorged larvae (originally mentioned as *L. paniculatum*) gathered with use of the black plate method in Thailand tested positive for *O. tsutsugamushi* [68].

***L. peromysci* Vercammen-Grandjean and Langston, 1976**

Parasitic larvae found on the white footed deer mouse *Peromyscus leucopus* Rafinesque, 1818 (Mammalia: Rodentia) in the USA (North Carolina) turned out positive for *Rickettsia* sp. and species very closely related to *R. conorii*, *R. felis* and *R. typhi* [64].

***L. rajasthanense* Fernandes and Kulkarni, 2003**

*Orientia tsutsugamushi*-positive larvae of this species fed on *R. rattus* captured in India [49].

***L. rubellum* Wang and Liao, 1984**

Listed among *O. tsutsugamushi* vectors in China [106].

***L. rupestre* Traub and Nadchatram, 1967**

Species found in mite pools positive for *O. tsutsugamushi* in Pakistan [14].

***L. scutellare* Nagayo, Miyagawa, Mitamura, Tamiya and Tenjin, 1921**

Species positive for *O. tsutsugamushi*, *R. typhi*, *R. felis*, *Rickettsia* sp. as well as pathogens most closely related to *R. akari* Huebner, 1946 and *R. australis* Philip, 1950 as well as a novel species *Candidatus* Rickettsia leptotrombidium. Host-searching larvae were gathered from plants and soil while parasites were collected from the bodies of *A. agrarius*, *A. agrarius chejuensis*, *A. speciosus speciosus*, the small Japanese field mouse *A. argenteus argenteus* (Temminck, 1844), *B. indica*, *Eothenomys kageus* Imaizumi, 1957, *M. montebelli*, *M. regulus*, *M. musculus*, *R. exulans*, *R. losea*, *R. norvegicus*, *R. tanezumi*, *T. triton*, *Urotrichus talpoides hondonis*, *C. lasiura* and *S. murinus*. Listed records originate from China, Japan, Malaysia, South Korea, Taiwan and Thailand [34,62,63,68,69,83,97,109,111,113,114,118,121,122,123,124,125,126].

***L. sialkotense* Vercammen-Grandjean and Langston, 1976**

Listed among *O. tsutsugamushi* vectors in China [106].

***L. subintermedium* (Jameson and Toshioka, 1954)**

Mentioned among *O. tsutsugamushi* vectors in India and Pakistan [14,103].

***L. taishanicum* Meng, Xue and Wen, 1983**

*Orientia tsutsugamushi*-positive larvae were collected from *M. musculus* and *R. norvegicus* in China. Among tested hosts, also *A. argarius* and *T. triton* were bacteria-positive [109,111].

***L. tosai* (Sasa and Kawashima, 1951)**

Species (originally mentioned as *Trombicula tosa*) tested positive for *O. tsutsugamushi*. Larvae fed on *M. montebelli* and *A. speciosus* in Japan [107,123]

***L. turdicola* Vercammen-Grandjean and Langston, 1976**

Associated with *O. tsutsugamushi*. Parasites collected from *T. glis* in Thailand [24].

***L. umbricola* Nadchatram and Dohany, 1980**

Host-questing larvae tested positive for *O. tsutsugamushi* in Malaysia. Collected with the method of black plates [83,127].

Remarks: unengorged larvae of *L. vivericola* Vercammen-Grandjean and Langston, 1976 found in Malaysia by Dohany et al. [128] were also reported as *O. tsutsugamushi*-associated, however, the subsequent revision of the material proved the first species determination wrong and larvae were then assigned to *L. umbricola* [127].

***L. wenense* Wu, Wen, Yang and Wu, 1982**

Listed among *O. tsutsugamushi* vectors in China [106,129]

***L. zetum* (Traub, Morrow and Lipovsky, 1958)**

*O. tsutsugamushi*-associated larvae (mentioned as *L. zeta*) were collected from the mice, including *A. agrarius*, in South Korea [34,116].

**Genus: *Lorillatum* Nadchatram, 1963**

***Lorillatum* sp.**

Genus representatives were reported to harbor *O. tsutsugamushi*. Larvae parasitized rodents from *R. rattus*-complex in Thailand [50].

**Genus: *Microtrombicula* Ewing, 1950**

***Microtrombicula* sp.**

Reported as positive for *Candidatus* Orientia chuto Izzard et al., 2010. Parasites collected from ‘*Micromys natalensis*’, in Kenya [130].

***M. chamlongi* Nadchatram and Kethley, 1974**

Unengorged larvae collected from the black plates spread over the ground tested positive for *O. tsutsugamushi*. Record from Thailand [68].

**Genus: *Miyatrombicula* Sasa, Kawashima and Egashira, 1952**

***M. kochiensis* Sasa, Kawashima and Egashira, 1952**

*Orientia tsutsugamushi*-associated species. Unengorged larvae were gathered from the ground and vegetation in Japan [69].

**Genus: *Neoschoengastia* Ewing, 1929**

***Neoschoengastia* sp.**

Listed in *Epidemiology and ecology of rickettsial diseases in the People’s Republic of China* as a genus characterized by low rate infection with *O. tsutsugamushi* [44].

**Genus: *Neotrombicula* Hirst, 1925**

***Neotrombicula* sp.**

Evidenced as positive for *Candidatus* Orientia chuto. Larvae were collected from ‘*Micromys natalensis*’, in Kenya. Reported also from Spain as associated with *R. felis* [120,131].

***N. autumnalis* (Shaw, 1790)**

Individuals of the European harvest mite were reported to contain *Anaplasma phagocytophilum* (Foggie 1949) (Pseudomonadota, Rickettsiales, Anaplasmataceae) (originally mentioned as *Ehrlichia phagocytophila*), *B. burgdorferi s.l.* Johnson et al., 1984, *B. garinii* Baranton et al., 1992, *B. valaisiana* Wang et al., 1997, *R. monacensis*, *R. helvetica*, *R. pavlovskyi* and *Rickettsia* sp. Infected larvae were collected from rodents (*M. glareolus*, *A. flavicollis*, *M. arvalis* and *A. sylvaticus*), birds (the Eurasian blackcap *Sylvia atricapilla* (Linnaeus, 1758) (Aves: Passeriformes)), soricomorphs (the white-toothed shrew *C. russula* (Hermann, 1780)), lizards (the common wall lizard *Podarcis muralis* (Laurenti, 1768), the Italian wall lizard *P. siculus* (Rafinesque, 1810) and the western green lizard *Lacerta bilineata* Daudin, 1802 (Reptilia: Squamata)) as well as from the vegetation. One case of infected nymph was reported too. Observations were made in Czech Republic, Germany, Italy, Russia, Slovakia, Spain and Ukraine [58,65,132,133,134,135,136].

Remarks: According to Stekolnikov et al. [18], all records concerning *N. autumnalis* should be thoroughly verified after proper mounting on microscopic slides, due to considerable resemblance of this species to *N. inopinata*. Proven misidentification was reported from Turkey.

***N. carpathica* Stekolnikov, 1996**

Reported as carrier of *B. garinii* and *B. valaisiana*. Larvae parasitized *S. atricapilla* in Czech Republic [134].

***N. inopinata* (Oudemans, 1909)**

Associated with *B. garinii*, *B. valaisiana* and *Rickettsia* spp. Parasitic larvae were gathered from *S. atricapilla* in Czech Republic and unengorged ones from the vegetation in Spain [134,137].

***N. japonica*** **(Tanaka, Kaiwa, Teramura and Kagaya, 1930)**

Frequently mentioned as *O. tsutsugamushi*-related species. Collected from wild rodents, mainly *A. agrarius*, *M. fortis*, ‘*C. triton*’ (most probably *T. triton*) and insectivorous *C. laisiura*, along with the host-questing larvae from the ground and vegetation. Records come from Japan, South Korea and Russia (Primorsky Krai) [28,34,63,69,115,117,120,138].

***N. microti* (Ewing, 1928)**

Mentioned as *O. tsutsugamushi*-positive species collected from rodents in Russia (Primorsky Krai) [139].

***N. mitamurai* (Sasa, Hayashi, Kumada and Teramura, 1950)**

Listed among *O. tsutsugamushi* carrying mites parasitizing rodents and shrews in Russia (Primorsky Krai) [28,116,138].

***N. nagayoi* (Sasa, Hayashi, Sato, Miura and Asahina, 1950)**

Mentioned as *O. tsutsugamushi*-positive species collected from rodents, including *A. agrarius*, in Russia (Primorsky Krai) and Korea [126,139].

***N. pomeranzevi* (Schluger, 1948)**

Larvae were reported as *O. tsutsugamushi*-associated. Parasites captured most probably from *M. rufocanus* (originally: *Clethrionomys rufocanus bedfordiae*) and *A. speciosus ainu* in Japan and Russia (Primorsky Krai) [110,137,139].

***N. sadoensis* Saito and Otsuru, 1959**

*Orientia tsutsugamushi*-positive representatives of the species were reported and taxonomically described from the Sado Island (Japan) [71].

***N. tamiyai* (Philip and Fuller, 1950)**

Mentioned (originally as *Trombicula tamiyai*) as *O. tsutsugamushi*-positive species collected from rodents and shrews in Russia (Primorsky Krai) [28].

***N. vulgaris* (Schluger, 1959)**

Larvae of the species revealed the presence of *R. helvetica* and *R. monacensis.* Gathered from *Rickettsia*-positive rodents: *M. glareolus*, *A. flavicollis*, *M. arvalis* and *A. sylvaticus* in Slovakia [58].

**Genus: *Odontocarus* Ewing, 1929**

***Odontocarus* sp.**

Unengorged larvae captured by the method of black plates tested positive for *O. tsutsugamushi*. Report from Thailand [68].

**Genus: *Parasecia* Loomis, 1966**

***Parasecia sp.***

*Rickettsia* sp. was detected in larvae of this genus parasitizing birds in Brazil [57]. 

**Genus: *Quadraseta* Brennan, 1970**

***Q. trapezoides* (Brennan and Jones, 1964)**

Parasite carrying a novel bacterium *Candidatus* Rickettsia colombianensi. Larvae were collected from the South American water rat *Nectomys squamipes* (Brants, 1827) (Mammalia: Rodentia) in Brazil [76].

**Genus: *Sauriscus* Lawrence, 1949**

***S. sandovali* (Hoffmann, 1947)**

Larvae (originally mentioned as *Tecomatlana sandovali*) taken from the sac-winged bat *Saccopteryx bilineata* (Temminck, 1838) (Mammalia: Chiroptera) tested positive for *C. burnetii.* Record from Panama [77].

**Genus: *Schoengastia* Oudemans, 1910**

***Schoengastia* sp.**

Larvae of this genus are known for association with *B. tamiae*. Tested parasites were obtained from *R. rattus*, *R. argentiventer*, *B. indica*, *B. savilei* and *M. cervicolor* in Thailand [55].

**Genus: *Schoengastiella* Hirst, 1915**

***Schoengastiella* spp.**

Genus representatives parasitizing *R. rattus* were evidenced for the presence of *O. tsutsugamushi.* Observation from India [49].

***S. ligula* Radford, 1946**

Individuals captured on *R. rattus* and *S. murinus* tested positive for *O. tsutsugamushi* in India. The association was mentioned also from Malaya and Pakistan [14,49,140].

**Genus: *Schoutedenichia* Jadin and Vercammen-Grandjean, 1954**

***Schoutedenichia* sp.**

Mites of the genus are reported to carry *O. tsutsugamushi* and *Rickettsia* sp. Bacteria-positive larvae were obtained from *R. tanezumi*, *B. savilei*, and *B. indica* in Thailand [23,47].

**Genus: *Trombewingia* Fonseca, 1955**

***T. bakeri* (Fonseca, 1955)**

Mite associated with a novel bacterium *Candidatus* Rickettsia colombianensi. Larvae were captured feeding on the montane grass mouse *Akodon montensis* Thomas, 1913 (Mammalia: Rodentia) in Brazil [76].

**Genus: *Trombiculindus* Radford, 1948**

***T. variaculum* (Traub and Nadchatram, 1967)**

*Orientia tsutsugamushi*-associated species, larvae of which were collected from *R. exulans* in Thailand [23].

**Genus: *Walchia* Ewing, 1931**

***Walchia* sp.**

*Orientia tsutsugamushi*-positive chiggers of this genus were obtained from *B. indica*. Thailand [23].

***W. chinensis* (Chen and Hsu, 1955)**

Known for carrying *O. tsutsugamushi*. Larvae taken mostly from *R. tanezumi flavipectus,* captured in China [44,141].

***W. kritochaeta* (Traub and Evans, 1957)**

Species tested positive for the scrub typhus etiologic agent. Parasitic larvae were collected from *R. exulans*, *R. tanezumi*, *B. indica*, *B. berdmorei* and the red spiny rat *Maxomys surifer* (Miller, 1900) in Thailand [23].

***W. masoni* (Asanuma and Saito, 1957)**

Larvae captured from wild hares (the Japanese hare *Lepus brachyurus* Temminck, 1845 (Mammalia: Lagomorpha)) in Japan were positive for bacterium related to *O. tsutsugamushi*, however, according to authors, the record is not fully confirmed [14,71].

***W. micropelta* (Traub and Evans, 1957)**

Larvae of the species contained genetic material of *O. tsutsugamushi*. Collected from *B. indica* and *M. surifer* in Thailand [23].

***W. minuscuta* (Chen, 1978)**

Parasitic larvae found on *M. surifer* in Thailand tested positive for *O. tsutsugamushi*. Species also known to harbor *Borrelia* sp. [23,40].

***W. ogatai* Sasa and Teramura, 1951**

Host-questing larvae gathered from the vegetation in Japan tested positive for *O. tsutsugamushi* [69].

***W. pacifica* (Chen and Hsu, 1955)**

*O. tsutsugamushi*-associated larvae were obtained from rodents, mainly, *A. agrarius*, *R. norvegicus* and *T. triton* in China [111,113,114].

**Figure 1:**
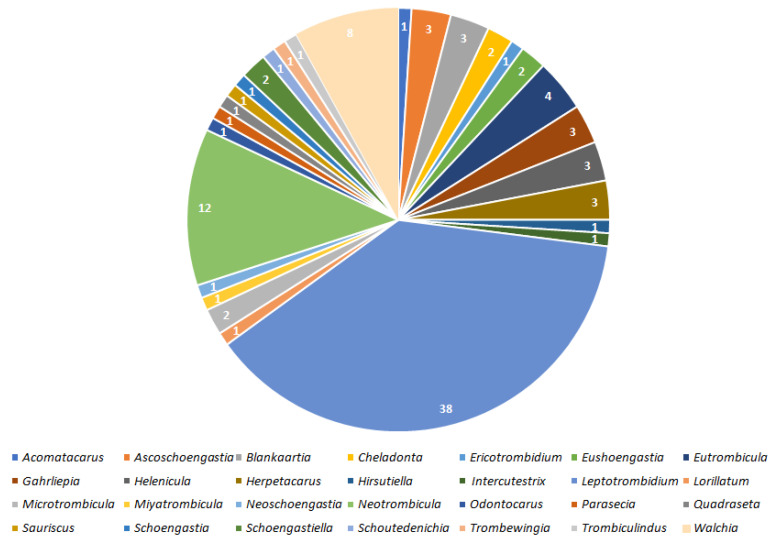
Numbers of species within 28 mite genera associated with bacterial taxa.

**Figure 2:**
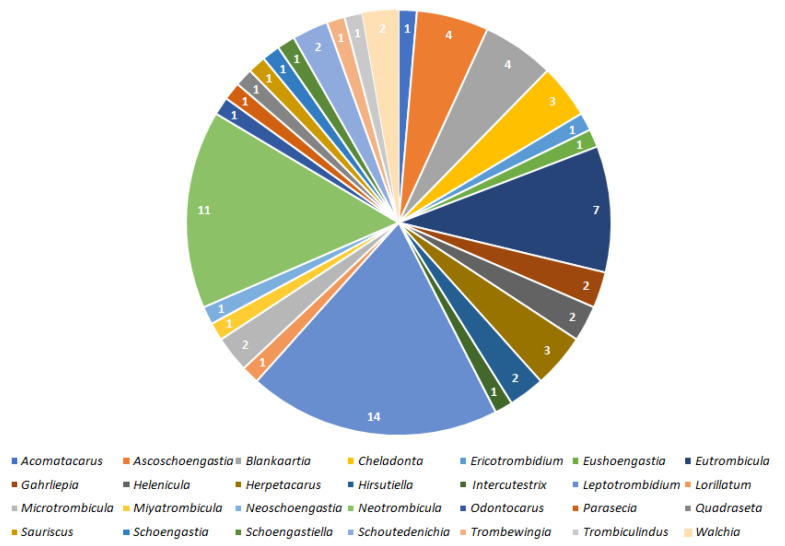
Numbers of bacterial taxa detected in 28 genera of mites.

The above records are graphically summarized in Figure 1 and Figure 2.

**Trombiculidae spp.**

Metagenomic analysis of rodent-associated chiggers collected in Thailand showed that undetermined chiggers were infected with the following pathogens: *Bartonella* spp., *Borrelia* spp., *Francisella* spp. Dorofe’ev 1947 (Pseudomonadota, Thiotrichales, Francisellaceae), *Leptospira* spp. Noguchi, 1917 (Spirochaetota, Leptospirales, Leptospiraceae) and *O. tsutsugamushi* [142]. Additionally, *Mycobacterium* sp. was detected in trombiculids parasitizing rodents and insectivores from Thai populations [40].

Pioneer survey on ectoparasites of rodents and related pathogens carried out in Saudi Arabia revealed the presence of *Borrelia* spp., *C. burnetii*-like bacterium and *Candidatus* Orientia chuto in trombiculids collected from the Eastern spiny mouse *Acomys dimidiatus* (Cretzschmar, 1826), the Yemeni mouse *Ochromyscus yemeni* (Sanborn and Hoogstraal, 1953), the king jird *Meriones rex* Yerbury and Thomas, 1895 (Mammalia: Rodentia) and *R. rattus* [143,144].

Research on pathogens associated with trombiculids parasitizing *A. agrarius* and *C. lasiura* in South Korea indicated mites’ association with *Rickettsia* sp., *R. akari*, *R. australis*, *R. conorii*, *R. felis*, *R. japonica* Uchida et al., 1992 and *R. typhi* [145].

**Figure 3:**
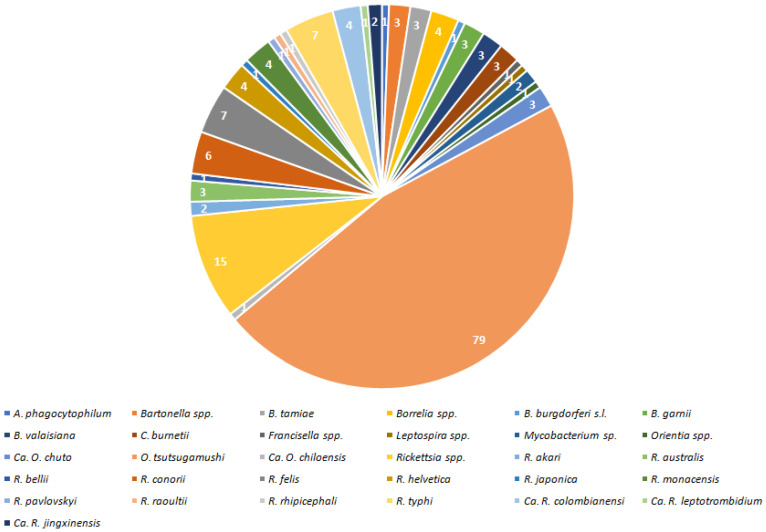
Incidence of particular bacterial pathogens in hereby listed Trombiculidae.

**Figure 4:**
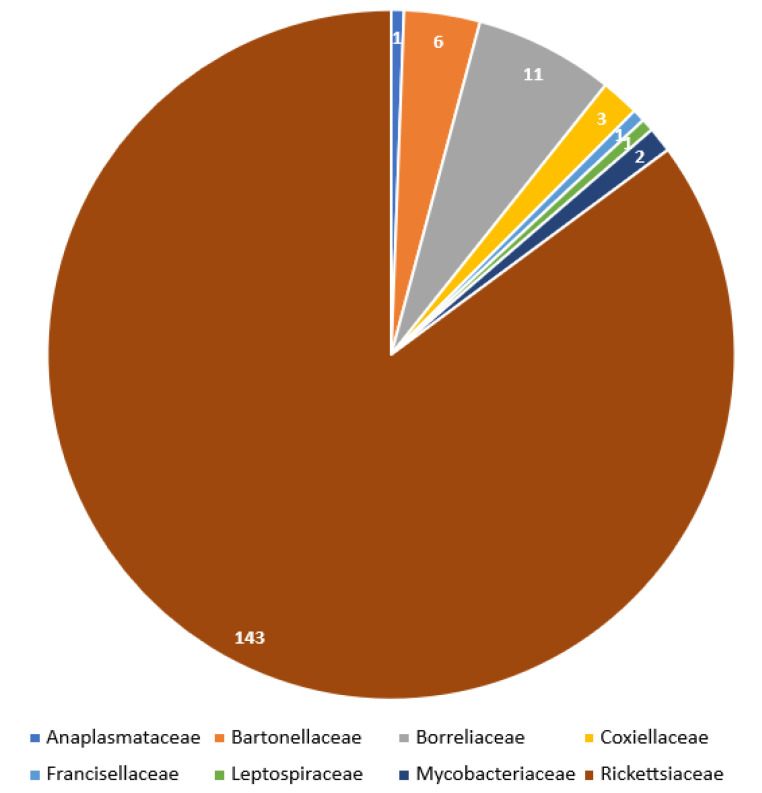
Incidence of associations between pathogens and listed trombiculids grouped by bacterial families.

Occurrence frequency of particular bacteria (genera and species) as well as bacterial families associated with Trombiculidae are illustrated in Figure 3 and Figure 4.

### 2.2. Species Lacking Authorities

***Leptotrombidium nangii***

***L. tachensis***

***L. waiganmensis***

The three taxa were listed in *Epidemiology and ecology of rickettsial diseases in the People’s Republic of China* and described as infected with *O. tsutsugamushi* at low rate [44].

Remarks: Species not listed in the reference checklists, even as synonyms.

***Neotrombicula shiraii***

Listed among *O. tsutsugamushi*-associated mites in Japan [10,71].

Remarks: This combination has not been found in the current literature, even as a synonym. Reports on ‘*Neoschoengastia shiraii*’, a parasite of birds occurring in Japan, can be found but without mention of scrub typhus bacterium [146]. 

## 3. Discussion

Results indicate that 100 species of Trombiculidae, representing 28 genera, are associated with 31 bacterial taxa from eight families, seven orders and three phyla (Figure 1, Figure 2, Figure 3 and Figure 4). Listed mites constitute around 3.3% of the whole family and occur in Palaearctic, Nearctic, Neotropical, Afrotropical, Oriental and Australian zoogeographic regions [2]. Apart from non-parasitic instars (i.e., host searching larvae swarming on the ground and vegetation and predatory deutonymphs), pathogen-positive individuals include ecto- and endoparasites of rodents (majority of cases), soricomorphs, scadents, lagomorphs, peramelemorphs, bats, passerine birds, reptiles and humans. Among listed genera, *Leptotrombidium* is characterized by the highest number of bacteria-positive species (38), followed by *Neotrombicula* (12) and *Walchia* (8) (Figure 1). This might be reflecting the fact that named genera are considerably numerous in species [2,86]. As for the number of bacteria associated with particular genera, again, *Leptotrombidium* spp. are positive for the highest quantity of pathogenic taxa (14), next is *Neotrombicula* (11) and *Eutrombicula* (7) (Figure 2). Rickettsiaceae is the dominant family detected in Trombiculidae. *Orientia tsutsugamushi* infection was confirmed in 79 cases, and is followed by *R. typhi* and *R. felis* (7 cases, each), to name only the most frequent rickettsial species. Considerable incidence also characterizes Borreliaceae—infection with *Borrelia* spp. was observed in 11 cases (Figure 3 and Figure 4). Hereby discussed data concern only non-artificial infections of mites originating from the natural environment. We excluded experimentally induced associations, such as acquisition of *B. garinii* and *C. burnetii* by larvae of the European harvest mite after parasitizing infected rodents under laboratory conditions, or obtaining *O. tsutsugamushi*-positive larvae of the common Northamerican chigger resulting from experimental feeding upon host with rickettsaemia [42,54,133]. Associations with viruses, for example, between Hantaan virus and *L. scutellare* [147,148], however very interesting, were omitted too.

Rickettsiales, the obligate intracellular parasites [66], are especially prevalent within chigger mites. With the highest probability, it is the result of the already described larval feeding mechanism (dissolving and ingesting cell contents from host’s epidermis and dermis) [16], combined with the affinity of rickettsial bacteria to reside in the connective tissue, including its external layers forming the skin [149,150,151,152]. Similar tendency is characteristic for *Borrelia* spp. spirochetes [153,154,155]. Producing the stylostome during food intake is a common feature of trombiculids, however, its length, width and wall structure is differentiated among genera [16,156]. Hase et al. proposed three types of stylostomes depending on the longitude (epidermal—the shortest, mesenchymal—the longest and a mix of the two) based on observations of *Leptotrombidium* spp feeding. Authors hypothesized that the canal structure might have been related to the mite’s ability to acquire bacteria, as larvae of *L. intermedium* (the only species to form epidermal stylostome) turned out to be pathogen-free [157]. This assumption was subsequently refuted as later studies revealed the association between *O. tsutsugamushi* and *L. intermedium*. Moreover, *Neotrombicula pomeranzevi*, *Miyatrombicula esoensis* (Sasa and Ogata, 1953) and *Kepkatrombicula desaleri* (Methlagl, 1928) produce the longest feeding canals, thus penetrating host tissues relatively deep, albeit the ability to ingest pathogens by the two latter species still awaits confirmation in laboratory tests. On the other hand, *H. zachvatkini* and *Leptotrombidium* spp. Create shorter canals which widen in time and were reported as bacteria-positive quite frequently (Figure 1 and Figure 2). *Cheladonta costulata*, known for parasitism entailing submergence of almost the entire body into host tissues and producing feeding tubes of very variable length, was capable of acquiring rickettsiae too [16,156]. Considering the fact that the current number of pathogen-associated trombiculids is 100, whereas formation of the stylostome has been examined in a fracture of this group, the relation between the structure of the feeding canal and acquisition of bacteria is still to be verified in the research involving more species. A common feature of the remaining bacterial genera detected in trombiculids (i.e., *Bartonella, Coxiella, Francisella*, *Leptospira*, *Mycobacterium*) is their natural presence in organisms of Rodentia and Soricomorpha [42,158,159,160,161,162], which are not only preferable hosts of chiggers in general but also were sources from which infected parasitic instars were collected. Named microorganisms, however, (unlike Rickettsiaceae and Borreliaceae) do not demonstrate high affinity to external layers of the connective tissue, but are reported to reside mostly in phagocytes, endothelium, erythrocytes and kidney cells as well as in soil and water [163,164,165,166,167,168]. This may potentially explain lower incidence of these genera in mites (Figure 3 and Figure 4). Inasmuch some bacterial species from the above genera can also dwell in the moist microhabitats (also preferred by many trombiculids), it cannot be excluded that their presence in mites might be a result of contamination.

A term *association* between mites and pathogens has been deliberately applied as vectorship, i.e., capability of effectively transmitting bacteria to humans (or other vertebrates) is not proven for all pathogen-positive chigger species and the presence of microorganisms may be resulting from ingesting dissolved host tissues (i.e., pathogen’s reservoir), especially when engorged larvae are preserved and tested shortly after being detached from the host. Confirmed vectors of *Orientia* spp., e.g., *L. deliense*, *L. akamushi*, *L. scutellare* or *H. antarctica*, meet the criteria formulated by Traub and Wisseman: natural infection with a pathogen, ability to infect a host (by feeding process, after being crushed on host’s body or with infected feaces), high prevalence in a given area and, tendency for parasitizing humans. The latter point regards of course only diseases plaguing people and it is not essential for the mechanism of vectorship as such. As Trombiculidae, unlike, e.g., Ixodidae or Macronyssidae, are parasitic once in a lifetime, to successfully transmit bacteria larvae have to acquire them via transstadial and transovarial transmission from the parental generation [14,54]. The latter two phenomena also have been analyzed mainly in *Leptotrombidium* spp. so far e.g., [19,20,21]. At the same time, observations of bacteria-positive, unengorged larvae from *Eutrombicula*, *Herpetacarus*, *Microtrombicula*, *Miyatrombicula*, *Neotrombicula*, *Odontocarus* and *Walchia* genera along with the infected deutonymphs and successful experimental infections, indicate that the possibility of effective pathogen transmission remains high in a variety of chigger mite species.

One should bear in mind, yet, that detailed research on vector competence of the particular mites species is often hindered by two issues. The first one is extremely varied numbers of mites from particular species in collected samples—ranging from thousands of individuals of the most common taxa, to single ones of the most infrequent (H.M. personal observation). This is illustrated by results of chigger collection in NW Russia wherein thousands of *H. zachvatkini* larvae were present in contrast to nine of *A. latyshevi* (Schluger, 1955) and one *N. absoluta* Schluger, 1966 larva. Significant disproportions in field-collected chiggers were also recorded during a survey performed in India—the most common species *L. deliense* and *L. insigne* Fernandes and Kulkarni, 2003 combined totalled 9408 larvae, while the rarest—*Walchia* sp. and *Schoutedenichia* sp. were 33 and single larva, respectively. Tamura et al., in turn, observed the following shares of mites collected in Japan: *L. pallidum* (56.6%), *L. scutellare* (13.6%), *L. fuji* (12.7%), *G. saduski* (10.5%), *N. japonica* (1.6%), *L. kitasatoi* (1.6%), *L. palpale* (1.4%), *L. intermedium* (1.1%), *L. miyazakii* Sasa, Sawada, Kano, Hayashi and Kumada, 1951 (0.1%), *L. miyajimai* (Fukuzumi and Obata, 1951) (0.3%), *N. tamiyai* (0.02%), *Eltonella ichikawai* Vercammen-Grandjean, 1965 (0.1%) and *C. ikaoensis* (0.1%) [92,169,170]. Although single specimens can be easily tested for the presence of bacteria, results based on small samples are not fully representative. Moreover, verification of transstadial and transovarial transmissions would require experimental rearing of a few mite generations based on a considerable number of chiggers, with consideration of their mortality [171]. The second problem is the correct identification of potential vector species. An example of this kind of impediment is already signalized misidentification between closely related *N. autumnalis* (considered the most common European chigger, however, absent in some countries (H.M. personal observation) and *N. inopinata* (species often determined after the proper identification of specimens previously assigned to ‘*N. autumnalis*’) [18]. Furthermore, Ponnusamy et al. have recently reported on problems with matching COI sequences of *Rickettsia*-positive *Eutrombicula* sp. and *Leptotrombidium* sp., previously determined upon morphological criteria, with corresponding data in the GeneBank using the BLAST tool [64]. This problem is not limited to chiggers inhabiting Nearctic, as the deficiency of reference sequences in the GeneBank is still noticeable (such data are available for *c.* 80 nominal species only). At the same time, obtaining COI sequences of *L. imphalum* and *L. chaigraiensis* led to separation of these species, formerly considered as one [86,88].

Identification difficulties should not suppress the research on pathogen transmission by Trombiculidae inasmuch the most accurate species determination can be achieved by simultaneous application of morphological and molecular tools, as it has been already implemented in some studies [88,172,173,174]. Moreover, this issue necessitates further exploration as bacterial diseases potentially transmitted by chiggers are largely negligent in parts of the globe where they are apparently absent, as it was emphasized by Weitzel et al. [39]. For example, not only is the scrub typhus present in the subantarctic Chile, but also its etiological agent turned out to be a novel *Orientia* species—*Candidatus* Orientia chiloensis. The pathogen was effectively transmitted to humans by *H. antarctica* [36,39,73,175]. Analogical instances of bush typhus bacterium, distinct from *O. tsutsugamushi* (i.e., *Candidatus* Orientia chuto), detected in Trombiculidae occurring outside the ‘tsutsugamushi triangle’ come from Kenya and Saudi Arabia. From the latter country, *O. chuto*-positive patient was reported as well [37,38,130,143]. Other proposed bacterial species (i.e., *Candidatus* rickettsia colombianesi, *Ca*. rickettsia jinxinensis and *Ca*. rickettsia leptotrombidium) harbored by Trombiculidae, along with the most recent and the first ever findings of rickettsiae in chiggers from North Carolina (USA) [64] only reaffirm the sense of continual survey on, otherwise unrecognized and hidden, pathogens and their transmission routes. Exploring the issue is also justified by non-decreasing importance of parasitic and bacterial zoonoses in general [42] and can be greatly supported by the highly effective technologies (e.g., RAPD-PCR fingerprinting or high throughput sequencing (HTS) related techniques such as DNA metabarcoding), application of which can result in further contributions to the hereby reviewed matter.

## 4. Materials and Methods

Google scholar, PubMed and Scopus databases were searched with *bacteria*, *chigger mites*, *detection*, *pathogen*, *spirochaetes*, *Trombiculidae*, *trombiculid* terms. Collected records cover almost 100 years (from 1924 to 2022) of the research on the association between chiggers and bacterial pathogens, detected by means of microscopic, culturing, serological and molecular examinations performed worldwide (Section 2.1). Testing techniques as well as the very history of studies on Trombiculidae-bacteria relation are summarized and thoroughly described in the literature [10,32].

Taxonomic nomenclature and systematics of Trombiculidae follow elaborations, taking into account morphological and molecular data [2,86,87,88,103]. Species authorities, scientific names as well as common names and systematics of vertebrates are given at first mention, according to Wilson and Reeder and IUCN Red List [176,177].

Species of Trombiculidae lacking authority and not present in the above reference sources are placed in a separate subsection (2.2) but are not included in plots and calculations.

## 5. Conclusions

The share of pathogen-associated trombiculids is low in comparison with the total number of nominal species comprising the Trombiculidae family. Nonetheless, so far revealed bacteria-positive mites are characterized by harboring differentiated bacterial species, vast geographical distribution and association with a variety of hosts. Moreover, the present summary also points at cases of relatively recently discovered novel bacterial species and localities wherein the discussed microorganisms were apparently absent to date. This knowledge, combined with the unwavering significance of zoonotic bacterioses and the recognized mechanisms of pathogens circulation in chigger populations, are premises that the actual number of Trombiculidae-bacteria associations is not limited to the cases presented. An assumption can be made that the continual microbiological testing of chiggers, especially when supported with the fast and highly effective technologies, will result in further findings.

## Data Availability

Not applicable.
